# Serum Procalcitonin and Peripheral Venous Lactate for Predicting Dengue Shock and/or Organ Failure: A Prospective Observational Study

**DOI:** 10.1371/journal.pntd.0004961

**Published:** 2016-08-26

**Authors:** Vipa Thanachartwet, Varunee Desakorn, Duangjai Sahassananda, Akanitt Jittmittraphap, Nittha Oer-areemitr, Sathaporn Osothsomboon, Manoon Surabotsophon, Anan Wattanathum

**Affiliations:** 1 Department of Clinical Tropical Medicine, Faculty of Tropical Medicine, Mahidol University, Bangkok, Thailand; 2 Information Technology Unit, Faculty of Tropical Medicine, Mahidol University, Bangkok, Thailand; 3 Department of Microbiology and Immunology, Faculty of Tropical Medicine, Mahidol University, Bangkok, Thailand; 4 Pulmonary and Critical Care Division, Department of Medicine, Phramongkutklao Hospital, Bangkok, Thailand; 5 Hospital for Tropical Diseases, Faculty of Tropical Medicine, Mahidol University, Bangkok, Thailand; 6 Pulmonary and Critical Care Division, Department of Medicine, Ramkhamhaeng Hospital, Bangkok, Thailand; Oregon Health and Science University, UNITED STATES

## Abstract

**Background:**

Currently, there are no biomarkers that can predict the incidence of dengue shock and/or organ failure, although the early identification of risk factors is important in determining appropriate management to reduce mortality. Therefore, we sought to determine the factors associated with dengue shock and/or organ failure and to evaluate the prognostic value of serum procalcitonin (PCT) and peripheral venous lactate (PVL) levels as biomarkers of dengue shock and/or organ failure.

**Methodology/Principal Findings:**

A prospective observational study was conducted among adults hospitalized for confirmed viral dengue infection at the Hospital for Tropical Diseases in Bangkok, Thailand between October 2013 and July 2015. Data, including baseline characteristics, clinical parameters, laboratory findings, serum PCT and PVL levels, management, and outcomes, were recorded on pre-defined case report forms. Of 160 patients with dengue, 128 (80.0%) patients had dengue without shock or organ failure, whereas 32 (20.0%) patients developed dengue with shock and/or organ failure. Using a stepwise multivariate logistic regression analysis, PCT ≥0.7 ng/mL (odds ratio [OR]: 4.80; 95% confidence interval [CI]: 1.60–14.45; *p* = 0.005) and PVL ≥2.5 mmol/L (OR: 27.99, 95% CI: 8.47–92.53; *p* <0.001) were independently associated with dengue shock and/or organ failure. A combination of PCT ≥0.7 ng/mL and PVL ≥2.5 mmol/L provided good prognostic value for predicting dengue shock and/or organ failure, with an area under the receiver operating characteristics curve of 0.83 (95% CI: 0.74–0.92), a sensitivity of 81.2% (95% CI: 63.6–92.8%), and a specificity of 84.4% (95% CI: 76.9–90.2%). Dengue shock patients with non-clearance of PCT and PVL expired during hospitalization.

**Conclusions/Significance:**

PCT ≥0.7 ng/mL and PVL ≥2.5 mmol/L were independently associated with dengue shock and/or organ failure. The combination of PCT and PVL levels could be used as prognostic biomarkers for the prediction of dengue shock and/or organ failure.

## Author Summary

Dengue is a major global health concern, particularly in tropical countries, and affects all age groups. Mortality rates among patients who have been hospitalized with severe dengue are 1.6–10.9%, and death in adults is mainly due to the development of dengue shock and organ dysfunction. In states of poor tissue circulation or shock, lactate is produced. Additionally, procalcitonin is a highly specific biomarker of systemic inflammation. Therefore, we assessed whether procalcitonin and peripheral venous lactate could be used to predict the incidence of dengue shock and/or organ failure in patients with dengue. Our study showed that a combination of serum procalcitonin levels ≥0.7 ng/mL and peripheral venous lactate levels ≥2.5 mmol/L at admission could discriminate between patients who did and did not develop shock and/or organ failure, with high sensitivity and specificity. These parameters may therefore be useful as prognostic biomarkers. Our results suggest that serum procalcitonin is indicative of an extensive early inflammatory response, which may occur during the systemic phase of dengue. Peripheral venous lactate may be produced as a result of the poor tissue circulation that precedes dengue shock. Our findings may help clinicians to predict dengue shock and/or organ failure earlier to reduce in-hospital mortality.

## Introduction

Dengue is the most important arthropod-borne viral disease, and it exerts a high burden on populations and public health systems in most tropical countries [1,2]. The incidence has dramatically increased during the last 50 years (by 30-fold) for all four dengue virus serotypes (DENV 1–4) in more than 100 countries, including those in Southeast Asia, Central and South America, the Western Pacific, Africa, and the Eastern Mediterranean [2,3]. A previous report estimated that 390 million people are infected with DENV per year worldwide, of which 96 million show clinical manifestations of dengue [4]. Clinical manifestations range from acute febrile illness to severe dengue, which is a life-threatening condition [1]. In-hospital mortality is observed among 1.6–10.9% of patients with severe manifestations of dengue, including dengue hemorrhagic fever and/or dengue shock syndrome [5–7].

The World Health Organization (WHO) has implemented a goal of reducing dengue mortality by at least 20% and morbidity by 25% by the year 2020 [2]. Early recognition of severe dengue would help clinicians achieve close monitoring and provide proper fluid resuscitation in order to prevent severe disease, which would reduce mortality and morbidity. The revised 2009 WHO case definition was introduced in order to improve early recognition of severe dengue by increasing awareness of warning signs [1]. However, a recent systematic review showed that the definition had a wide range of sensitivity (59–98%) and specificity (41–99%) in the prediction of severe dengue [8].

The pathophysiology of severe dengue is complex, and involves an interplay of host immune and genetic factors with virulent strains of DENV [9,10]. The critical phase of severe dengue usually occurs as viremia declines [1]. DENV replication occurs within cells, particularly hepatocytes, monocytes, and macrophages, during systemic infection and the immune-mediated response following DENV infection, which is proportional to the viral load [11,12]. Immune-mediated pathogenesis has been considered a major cause of the increased vascular permeability of endothelial cells, leading to plasma leakage [9–11]. Delayed recognition and improper management of patients with plasma leakage can lead to shock and/or organ failure [11].

The prevalence of dengue shock among adults is approximately 18%, and it is the most common cause of death from DENV [13]. A previous systematic review and meta-analysis showed that several clinical factors, including age, female sex, neurological signs, nausea/vomiting, abdominal pain, gastrointestinal bleeding, hemoconcentration, ascites, pleural effusion, hypoalbuminemia, hypoproteinemia, hepatomegaly, high levels of aspartate aminotransferase (AST) and alanine aminotransferase (ALT), abnormal coagulators, primary/secondary infection, and DENV-2, were independently associated with the development of dengue shock [13].

Procalcitonin (PCT) is a functional immune modulating protein consisting of 114–116 amino acids, and is currently used as a novel biomarker for diagnostic and prognostic purposes [14]. PCT is produced and released into the bloodstream in response to infection and/or inflammation in various tissues. In particular, hepatocytes and peripheral blood mononuclear cells are potent PCT secretors [15]. A recent systematic review and meta-analysis showed that PCT was a useful biomarker for the early diagnosis of sepsis in critically ill patients, with a sensitivity and specificity of 77% and 79%, respectively [16]. The area under the receiver operating characteristics curve (AUROC) was 0.85, indicating moderate diagnostic accuracy [16]. In Southeast Asia, an endemic area for tropical infectious diseases, the AUROC for discrimination between bacterial and viral infections using PCT was 0.74, which was also indicative of moderate diagnostic accuracy [17]. Of the patients with dengue, 72% had a PCT level ≥0.1 ng/mL and 25% had a PCT level ≥0.5 ng/mL, which was higher than that of patients with influenza (34% at a PCT level ≥0.1 ng/mL and 16% at a PCT level ≥0.5 ng/mL [17]. Previous reports have also shown that PCT levels in patients with sepsis are associated with the severity of organ dysfunction [18], and that PCT could be used as a prognostic marker for discrimination between patients with and without septic shock, in addition to survival [19]. A previous study showed that PCT levels on admission were significantly higher among patients who died following infection with the 2009 H1N1 strain of influenza, compared with those who survived (14.5 vs. 1.7 ng/mL) [20]. In addition, arterial or venous lactate may be used as a biomarker for tissue hypoperfusion, regardless of organ failure or shock, particularly among patients with sepsis [21]. Our previous prospective study showed that peripheral venous lactate (PVL) concentration was independently associated with severe dengue [22].

In clinical practice, it can be difficult to identify the early stages of dengue shock and/or organ failure using clinical data. PCT and/or PVL may provide a superior prognostic method for predicting dengue severity at the time of hospital admission, particularly in the identification of patients at high risk of developing dengue shock and/or organ failure. At present, there have been no studies assessing the capacity of PCT and/or PVL to predict dengue shock and/or organ failure. Thus, we hypothesized that PCT and/or PVL may discriminate between patients who develop dengue shock and/or organ failure and those who do not. Therefore, we undertook a prospective observational study among hospitalized adults with dengue and determined the factors associated with dengue shock and/or organ failure. The prognostic values of PCT and PVL as biomarkers for predicting dengue shock and/or organ failure were evaluated.

## Methods

### Ethical considerations

The study design was approved by the Ethics Committee of the Faculty of Tropical Medicine, Mahidol University in Bangkok, Thailand. The Strengthening the Reporting of Observational Studies in Epidemiology (STROBE) statement (S1 Checklist) and the Standards for the Reporting of Diagnostic (STARD) accuracy (S2 Checklist) were followed in this study [23,24]. Patients aged ≥15 years with clinical dengue, defined as acute fever and ≥2 of the following symptoms were included: 1) headache, 2) ocular pain, 3) myalgia, 4) arthralgia, 5) rash, 6) a positive tourniquet test (≥20 petechiae per square inch), or 7) leukopenia (white blood cell [WBC] counts <5.0 × 10^3^ cells/μL). Patients had been admitted to hospital for treatment, and the broad criteria allowed physicians to invite all potential patients to participate in the study at the outpatient and emergency department. Written informed consent was obtained from all patients, or the patient's guardians if the patient was 15–18 years old, before participation in the study.

### Study design and population

This prospective observational study was performed among patients who were admitted to the Hospital for Tropical Diseases (Faculty of Tropical Medicine, Mahidol University in Bangkok, Thailand) between October 2013 and July 2015. The inclusion criteria were (i) age ≥15 years, (ii) clinical dengue, and (iii) confirmed dengue viral infection by reverse-transcriptase polymerase chain reaction (RT-PCR) from a serum sample obtained at admission, and/or positive micro-neutralization test results from serum samples obtained at admission and 2 weeks after admission, and/or dengue-specific immunoglobulin M (IgM) and immunoglobulin G (IgG) detected using enzyme-linked immunosorbent assays (ELISAs) in paired serum samples taken at admission and 2 weeks after admission. Patients with an underlying medical illness, mixed infection, current pregnancy, current use of any non-topical antibiotic, or current fluid therapy were excluded from this study.

Laboratory tests were conducted at admission, including a complete blood count and blood chemistry assessment, and samples for the measurement of PCT and PVL were collected. Blood samples for PCT and PVL analysis were collected every 24 h until the patient exhibited a body temperature of <37.8°C for 48 h. Treating physicians and investigators were blinded to the PCT and PVL results. All patients received standard management from their treating physicians, according to the 2009 WHO guidelines for dengue [1]. In order to exclude other infections, two blood samples for microbiological cultures were obtained, urinalysis was performed, and plain radiography of the chest was routinely performed at admission. Diagnostic tests for other infectious diseases were also performed when indicated by clinical findings at admission or during hospitalization. Dengue severity and outcomes were summarized on discharge. All patient data, including baseline characteristics, clinical parameters, laboratory findings, management, and outcomes, were recorded on a pre-defined case report form. At a 2-week follow-up appointment, blood samples were collected for complete blood counts and serum creatinine assessment. Subsequent follow-up was required within the following 2 months until the laboratory results reached reference ranges in order to serve as a baseline.

### Case definitions for dengue

The WHO 2009 dengue definition was used to classify dengue shock and organ failure in this study [1]. Dengue shock was defined as plasma leakage with shock. Plasma leakage was defined as ≥20% increase in hematocrit above baseline or clinical fluid accumulation manifested by pleural effusion, ascites, or serum albumin <3.5 g/dL. Shock was defined as (1) a rapidly weak pulse with pulse pressure <20 mmHg, or (2) a systolic blood pressure of <90 mmHg with tissue hypoperfusion evidenced by one of the following criteria: (i) decreased urine output (<0.5 mL/kg/h), (ii) impaired consciousness, (iii) AST >1000 IU/L, (iv) ALT >1000 IU/L, (v) cold skin, or (vi) clammy skin. Organ failure was defined as the presence of one of the following criteria: (i) respiratory distress (a respiratory rate of ≥24 breaths/min with <95% oxygen saturation in room air and/or the need for oxygen therapy), (ii) serum creatinine increased ≥3-fold from baseline, (iii) AST >1000 IU/L, (iv) ALT >1000 IU/L, (v) myocarditis, (vi) encephalitis, or (vii) spontaneous gastrointestinal bleeding requiring blood transfusion.

The WHO 2009 dengue definitions for warning signs (WSs) were also used in this study; WSs included (1) abdominal pain; (2) vomiting; (3) clinical fluid accumulation defined as the presence of pleural effusion determined by plain radiography of the chest or a serum albumin level <3.5 g/dL; (4) lethargy; (5) a liver span of >15 cm; (6) bleeding from a mucosal area, including the nose, gums, gastrointestinal tract, or vagina; and (7) an increase in hematocrit of 2% above the sex-specific reference range for a healthy Thai adult with a platelets of ≤100 × 10^3^/μL.

### Reverse-transcriptase polymerase chain reaction

Dengue viral RNA was detected from patient serum at admission using a two-step PCR method, as described by Lanciotti *et al*. [25], and modified using the methods of Reynes *et al*. [26]. Viral RNA was detected from acute serum samples using a PureLink Viral RNA/DNA Mini Kit (Invitrogen, Grand Island, NY, USA), according to the manufacturer’s instructions.

### Micro-neutralization test

Serum samples collected at admission and 2 weeks after admission were assayed for serotype-specific DENV using the micro-neutralization test described by Vorndam *et al*. [27], with the slightly modified protocol of Putnak *et al*. [28]. The micro-neutralization test based on the principle of the plaque reduction neutralization test was used to measure serotype specific anti-DENV neutralizing antibodies against all 4 serotypes. Serum samples were tested in triplicate and sera were serially diluted 2-fold from 1:20 to 1:5120 in a 96-well microplate. Each microplate included media only (negative control), a virus control and sera of known specific DENV serotypes (positive controls). The average number of virus foci were counted, and only assays with a virus control in the range of 50–60 foci per well and a media only control with no foci were included. For control sera of known DENV serotypes, at least 50% inhibition by viral replication was required (25–30 foci per well), compared with the virus control. The virus neutralization titer was defined as the reciprocal of the serum dilution providing 50% inhibition of viral replication compared with the virus control. A positive serotype specific anti-DENV test was defined as a 4-fold rise in neutralizing antibody titer in paired samples for 1 of the 4 DENV serotypes.

### Dengue viral infection serology

All sera collected at admission and 2 weeks after admission were tested using four separate capture ELISA assays for IgM and IgG against dengue virus and Japanese encephalitis virus, as described by Innis *et al*. [29]. The assay was performed using serum samples diluted 1:100. Assay results for test samples were expressed as units calculated by the following formula: units = 100 × (A_492_test sample–A_492_NS)/(A_492_PS–A_492_NS), where A_492_ was an absorbance at 492 nm, NS was a normal human serum negative standard, and PS was pooled sera from flavivirus infected patients. Both acute and convalescent sera were used for the assay. Only sera with either anti-dengue IgM or anti-Japanese encephalitis IgM levels ≥40 units were evaluated. To discriminate between dengue and other flavivirus infections, we determined the ratio of dengue IgM to Japanese encephalitis virus IgM, with a ratio ≥1.0 indicating dengue virus infection and a ratio <1.0 indicating other flavivirus infection. To discriminate primary from secondary dengue infection, the ratio of anti-dengue IgM to anti-dengue IgG was also calculated, with a ratio ≥1.8 indicating primary dengue infection and a ratio <1.8 indicating secondary dengue infection. This cut-off value was applied for either acute or convalescent samples as long as either an anti-dengue IgM or anti-dengue IgG response could be detected. Pooling the results for both acute and convalescent sera using the same cut-off value allowed more accurate classification of primary and secondary dengue.

### Measurement of serum procalcitonin

PCT was measured using an electrochemiluminescence method (Elecsys BRAHMS PCT, Roche Diagnostic, Mannheim, Germany) according to the manufacturer’s instructions using a Cobas e 411 immunoassay analyzer (Roche Diagnostic, Mannheim, Germany). Prior to assessment, frozen serum samples were stored at –80 °C by laboratory personnel blinded to patient status. The detection limit for the PCT assay was 0.02 ng/mL. The coefficients of variation for low and high concentrations were 1.7% and 1.4%, respectively.

### Measurement of peripheral venous lactate

Blood samples were collected from a vein in an upper extremity without the use of a tourniquet. A 2 mL blood sample was collected into a vacutainer tube containing sodium fluoride, immediately placed on ice, sent to the laboratory, and analyzed for lactate within 10 min. Lactate levels were measured by a colorimetric assay with an enzymatic reaction using an auto-analyzer (Roche/Hitachi Cobas C Systems, USA), according to the manufacturer’s protocol. The laboratory personnel were blinded to the sample sources. The coefficient of variation for the assay in our laboratory was 1.1%.

### Sample size calculation

A previous prospective study at the Hospital for Tropical Diseases (Bangkok, Thailand) indicated that the incidence of dengue shock and/or organ failure was 21.0% among hospitalized adults with dengue [30]. Based on this information, we calculated that a sample size of at least 122 patients was needed for this study, using a specificity of 90% with a confidence interval (CI) of ±6%.

### Statistical analysis

All data were analyzed using SPSS software (version 18.0; SPSS Inc., Chicago, IL). Numerical variables were tested for normality using Kolmogorov-Smirnov tests. Variables with non-normal distribution were summarized as medians and interquartile ranges (IQRs), and were compared using Mann-Whitney *U* tests for two-group comparisons. Categorical variables were expressed as frequencies and percentages, and were analyzed using chi-squared or Fisher’s exact tests, as appropriate. A univariate logistic regression analysis was performed with each potential factor included as an independent variable, and the presence or absence of dengue shock and/or organ failure as the dependent variable. Any variable with a *p*-value ≤0.2 was considered potentially significant and was further analyzed in a stepwise multivariate logistic regression analysis using a backward selection method for determining significant independent factors. The optimal cut-off values of factors predictive of dengue shock and/or organ failure were determined using ROC curves. Prognostic parameters were evaluated using 2 × 2 tables, and 95% CIs were calculated to determine sensitivity, specificity, negative predictive value (NPV), positive predictive value (PPV), positive likelihood ratio (LR+), and negative likelihood ratio (LR–). The optimal PCT and PVL cut-off values were then combined in a single “bioscore”, as described by Gibot *et al*, 2012 [31]. The bioscore attributed one point per biomarker with a value above or equal to the optimal cut-off value. The bioscore was defined as 0 (both biomarkers below their respective cut-off value), 1 (any one of the two biomarkers above/equal to the cut-off value), or 2 (both biomarkers above/equal to the cut-off value). The bioscore was then further tested for prognostic value in predicting dengue shock and/or organ failure by logistic regression analysis. All tests of significance were two-sided, with a *p*-value <0.05 indicating statistical significance.

## Results

### Study population

**Fig 1:**
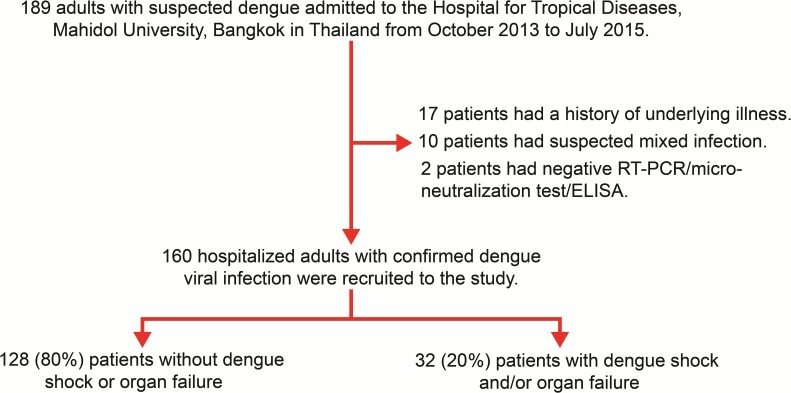
Flow diagram showing the recruitment of study patients. ELISA, enzyme-linked immunosorbent assay; RT-PCR, reverse-transcriptase polymerase chain reaction.

A total of 189 adults with suspected dengue were admitted to the Hospital for Tropical Diseases (Bangkok, Thailand) between October 2013 and July 2015. Of 189 hospitalized adults with suspected dengue viral infection, 29 patients were excluded due to an underlying illness (17 patients, 58.6%), mixed infection (10 patients, 34.5%), or a negative RT-PCR/micro-neutralization/ELISA for dengue (2 patients, 6.9%). Thus, 160 hospitalized adults with confirmed dengue viral infection were finally recruited for this study. Among the 160 patients, 32 (20.0%) patients had dengue shock (23 patients [71.9%]) and/or organ failure (26 patients [81.2%]), whereas 128 (80.0%) patients had dengue without shock or organ failure (Fig 1). In the 26 patients with organ failure, respiratory distress (11 patients [42.3%]), AST levels >1000 IU/L and/or ALT >1000 IU/L (9 patients [34.6%]), serum creatinine concentration ≥3-fold greater than baseline (6 patients [23.1%]), spontaneous gastrointestinal bleeding requiring blood transfusion (6 patients [23.1%]), myocarditis (4 patients [15.4%]), and encephalitis (3 patients [11.5%]) were observed.

### Comparison of baseline characteristics and clinical and laboratory parameters between dengue patients with and without shock and/or organ failure

At admission, patients with dengue shock and/or organ failure were significantly more likely to have a longer duration of fever (*p* = 0.031), skin bleeding (*p* = 0.012), mucosal bleeding (*p* <0.001), vomiting (*p* = 0.024), a liver span of >15 cm (*p* = 0.001), decreased breathing sounds (*p* <0.001), and increased respiratory rate (*p* = 0.010). When numerical parameters were categorized, patients aged >40 years (*p* = 0.023), with a fever duration ≥5 days (*p* = 0.041), respiratory rate ≥24 breaths/min (*p* = 0.005), mean arterial pressure <70 mmHg (*p* = 0.030), or pulse pressure <30 mmHg (*p* = 0.005) were more likely to have dengue shock and/or organ failure (Table 1 and S1 Table).

**Table 1:** Baseline characteristics and clinical parameters at admission among 160 hospitalized adults with dengue.

Characteristic	With dengue shock and/or organ failure (n = 32) n (%)	No dengue shock or organ failure (n = 128) n (%)	*p*-value
Age >40 years	11 (34.4)	19 (14.8)	0.023
Fever ≥5 days	19 (59.4)	48 (37.5)	0.041
Skin bleeding	25 (78.1)	66 (51.6)	0.012
Mucosal bleeding	25 (78.1)	52 (40.6)	<0.001
Vomiting	22 (68.8)	57 (44.5)	0.024
Liver span >15 cm	22 (68.8)	44 (34.4)	0.001
Respiratory rate ≥24 breaths/min	11 (34.4)	15 (11.7)	0.005
Pulse pressure <30 mmHg	11 (34.4)	15 (11.7)	0.005
Mean arterial pressure <70 mmHg	4 (12.5)	3 (2.3)	0.030

**Fig 2:**
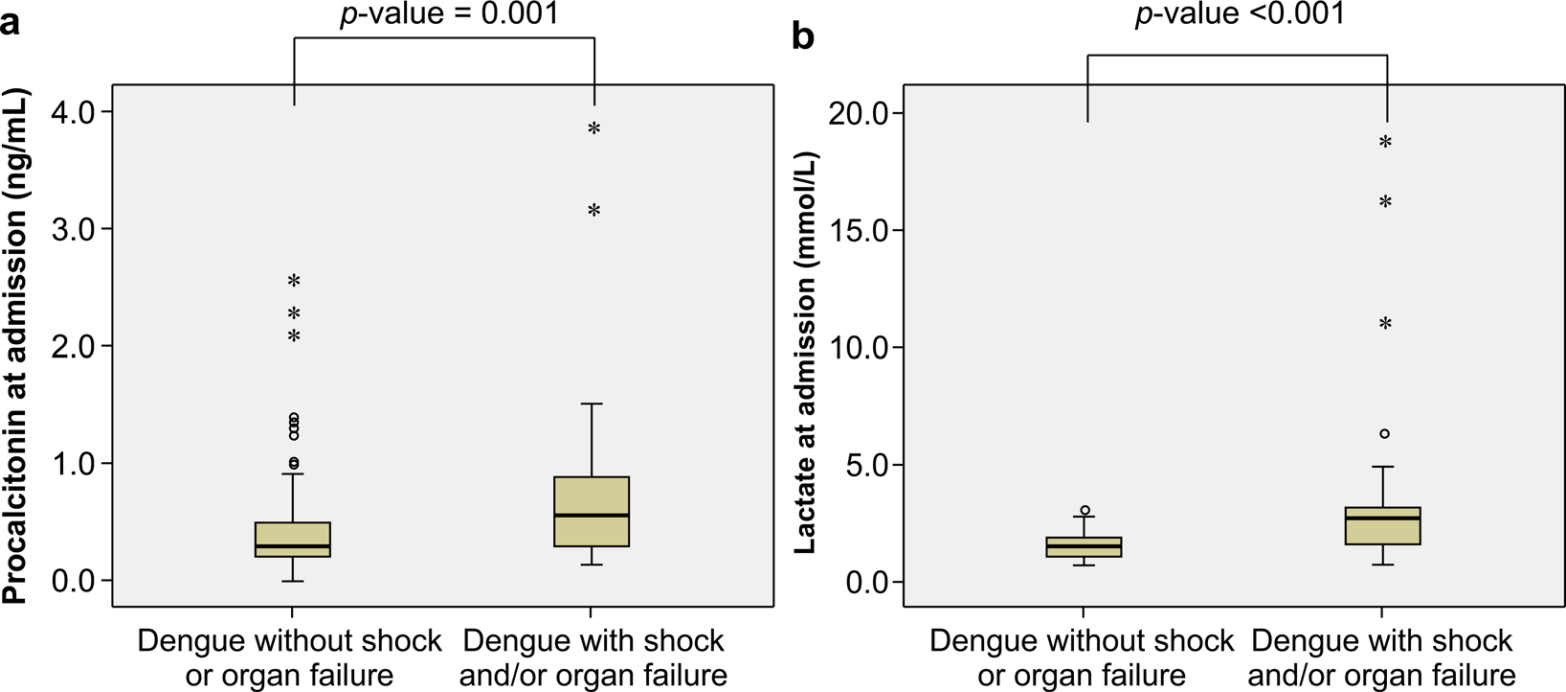
Serum procalcitonin and peripheral venous lactate at admission in dengue patients. (A) Serum procalcitonin levels among patients with and without dengue shock and/or organ failure. (B) Peripheral venous lactate levels among patients with and without dengue shock and/or organ failure. Data are presented as box and whisker plots with median (horizontal line), interquartile range (box), maximum value within 1.5 of interquartile range (whiskers), outliers (circles), and extreme outliers (asterisks).

Regarding laboratory parameters (Table 2 and S2 Table), patients with dengue shock and/or organ failure had significantly higher hemoglobin concentrations (*p* = 0.045), increased hematocrit values above baseline (*p* <0.001), higher WBC counts (*p* = 0.044), higher absolute bands (*p* = 0.022), higher absolute atypical lymphocyte counts (*p* = 0.007), higher AST levels (*p* <0.001), higher ALT levels (*p* <0.001), higher PCT levels (*p* = 0.001), and higher PVL levels (*p* <0.001) (Fig 2). However, patients with dengue shock and/or organ failure had significantly lower platelet counts (*p* <0.001) and albumin levels (*p* <0.001). When laboratory parameters were categorized based on the reference ranges (Table 2), patients with WBC counts >5.0 × 10^3^ cells/μL (*p* = 0.004), absolute bands >200 cells/μL (*p* = 0.049), absolute atypical lymphocyte counts >300 cells/μL (*p* = 0.006), AST >120 IU/L (*p* = 0.002), ALT >120 IU/L (*p* = 0.002), PCT ≥0.7 ng/mL (*p* = 0.002), and PVL ≥2.5 mmol/L (*p* <0.001) were more likely to have dengue shock and/or organ failure. In addition, patients with platelet counts <50.0 × 10^3^ cells/μL (*p* = 0.012) and albumin <3.5 g/dL (*p* = 0.001) were also more likely to have dengue shock and/or organ failure.

**Table 2:** Laboratory parameters categorized based on reference ranges among 160 hospitalized adults with dengue.

Characteristic	With dengue shock and/or organ failure	No dengue shock or organ failure	*p*-value
	(n = 32) n (%)	(n = 128) n (%)	
WBC >5.0 × 10^3^ cells/μL	15 (46.9)	26 (20.3)	0.004
Absolute bands >200 cells/μL	16 (50.0)	38 (29.7)	0.049
Absolute atypical LYM >300 cells/μL	17 (53.1)	33 (25.8)	0.006
Platelet counts <50.0 × 10^3^/μL	14 (43.8)	26 (20.3)	0.012
Albumin <3.5 g/dL	8 (25.0)	5 (3.9)	0.001
AST >120 IU/L	21 (65.6)	43 (33.6)	0.002
ALT >120 IU/L	15 (46.9)	24 (18.8)	0.002
Procalcitonin ≥0.7 ng/mL	13 (40.6)	18 (14.1)	0.002
Lactate ≥2.5 mmol/L	18 (56.3)	5 (3.9)	<0.001

ALT, alanine aminotransferase; AST, aspartate aminotransferase; LYM, lymphocytes; WBC, white blood cell counts.

Assessment of patient management and outcomes during hospitalization demonstrated that a significant proportion of patients with dengue shock and/or organ failure received albumin as fluid resuscitation (*p* <0.001) and antibiotics (*p* = 0.017). Of the 32 patients with dengue shock and/or organ failure, 4 (12.5%) received mechanical ventilation, 3 (9.4%) received renal replacement therapy, and 2 (6.2%) received vasopressors. Patients with dengue shock and/or organ failure had significantly longer durations of hospitalization (*p* = 0.006). However, only two patients expired during hospitalization, both due to multi-organ failure (S2 Table).

### Univariate and multivariate analyses for the prediction of dengue shock and/or organ failure

A univariate logistic regression analysis was used to determine which of the baseline characteristics, clinical parameters, and laboratory findings were associated with the occurrence of dengue shock and/or organ failure. All clinical factors potentially associated with the occurrence of dengue shock and/or organ failure were included in the univariate logistic regression analysis. The following variables were identified as clinical parameters associated with dengue shock and/or organ failure: (1) age >40 years, (2) fever duration ≥5 days, (3) absolute bands >200 cells/μL, (4) absolute atypical lymphocyte counts >300 cells/μL, (5) PCT ≥0.7 ng/mL, and (6) PVL ≥2.5 mmol/L (Table 3).

**Table 3:** Univariate analysis for the prediction of dengue shock and/or organ failure using clinical and laboratory parameters.

Characteristic	n	Odds ratio (95% CI)	*p*-value
Age >40 years	160	3.00 (1.25–7.22)	0.014
Fever ≥5 days	160	2.44 (1.10–5.37)	0.027
Absolute bands >200 cells/μL	160	2.37 (1.08–5.22)	0.032
Absolute atypical LYM >300 cells/μL	160	3.26 (1.47–7.26)	0.004
Procalcitonin ≥0.7 ng/mL	160	4.15 (1.76–9.92)	0.001
Lactate ≥2.5 mmol/L	160	31.63 (10.17–98.36)	<0.001

CI, confidence interval; LYM, lymphocytes.

All parameters with a *p*-value ≤0.2 in the univariate logistic regression analysis were then further analyzed by a stepwise multivariate logistic regression analysis using a backward selection method, in order to determine the independent factors significantly associated with the occurrence of dengue shock and/or organ failure. The following clinical and laboratory parameters were found to be independently associated with the occurrence of dengue shock and/or organ failure: (1) PCT ≥0.7 ng/mL (odds ratio [OR]: 4.80; 95% CI: 1.60–14.45; *p* = 0.005) and (2) PVL ≥2.5 mmol/L (OR: 27.99, 95% CI: 8.47–92.53; *p* <0.001) (Table 4). The two biomarkers PCT ≥0.7 ng/mL and PVL ≥2.5 mmol/L were assessed as a combined bioscore using a logistic regression model to evaluate the prognostic capacity in predicting the occurrence of dengue shock and/or organ failure. Higher bioscores were associated with increased occurrence of dengue shock and/or organ failure, with ORs of 22.23 (95% CI 7.85–63.00) and 30.00 (95% CI 5.76–156.31) for a bioscore 1 and 2, respectively (*p* <0.001) (Table 4).

**Table 4:** Multivariate logistic regression analysis for the prediction of dengue shock and/or organ failure using laboratory parameters.

Characteristic	n	Odds ratio (95% CI)	*p*-value
Procalcitonin ≥0.7 ng/mL	160	4.80 (1.60–14.45)	0.005
Lactate ≥2.5 mmol/L	160	27.99 (8.47–92.53)	<0.001
Combined procalcitonin and lactate (bioscore)			
0	114	1.00 (Reference)	
1	38	22.23 (7.85–63.00)	<0.001
2	8	30.00 (5.76–156.31)	<0.001

CI, confidence interval.

### Prognostic value of serum procalcitonin and peripheral venous lactate for predicting dengue shock and/or organ failure

**Fig 3:**
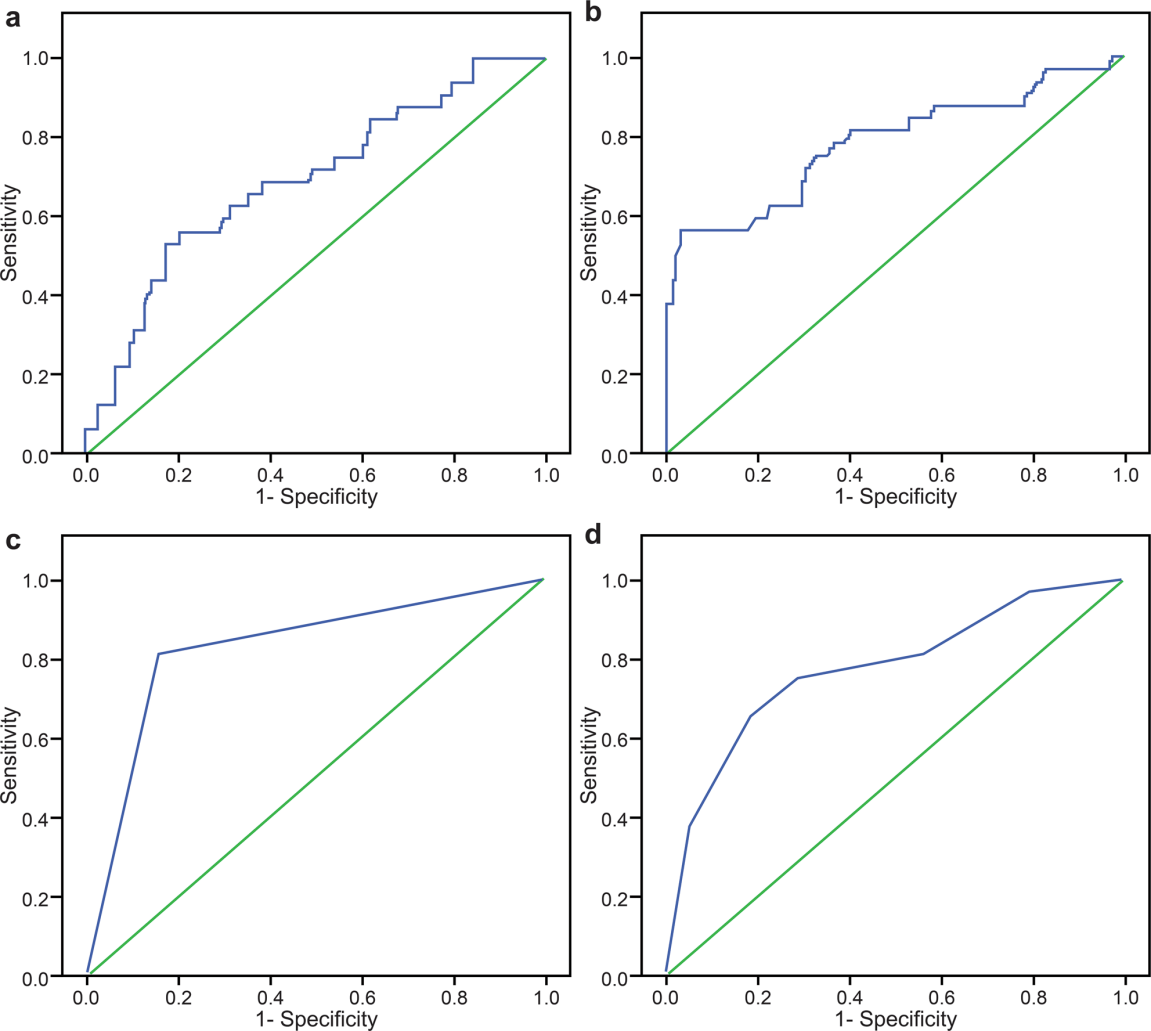
Receiver operating characteristic curves for serum procalcitonin and peripheral venous lactate in the prediction of dengue shock and/or organ failure at admission. (A) The area under the receiver operating characteristic curve (AUROC) for serum procalcitonin at admission was 0.69 (95% confidence interval [95% CI]: 0.59–0.80). (B) The AUROC for peripheral venous lactate at admission was 0.78 (95% CI: 0.68–0.88). (C) The AUROC for a combined bioscore at admission was 0.83 (95% CI: 0.74–0.92). (D) The AUROC for the number of warning signs at admission was 0.77 (95% CI: 0.68–0.87).

The AUROC for PCT in the prediction of dengue shock and/or organ failure was 0.69 (95% CI: 0.59–0.80) (Fig 3A). The AUROC for PVL in the prediction of dengue shock and/or organ failure was 0.78 (95% CI: 0.68–0.88) (Fig 3B). The prognostic values of PCT and PVL at admission for predicting dengue shock and/or organ failure are shown in Table 5. The sensitivities for nearly all PCT and PVL categories were low, except for PVL ≥1.5 mmol/L. The specificities for PCT and PVL categories were high, except for PCT ≥0.5 ng/mL and PVL ≥1.5 mmol/L. The PPVs and LR+ values for PCT and PVL categories were low, except for PVL ≥2.5 mmol/L and ≥3.0 mmol/L. The NPVs for all PCT and PVL categories were high. The LR–values for PCT and PVL were high, indicating possible prediction of dengue shock and/or organ failure.

**Table 5:** The prediction of dengue shock and/or organ failure at admission using serum procalcitonin and peripheral venous lactate.

Cut-off value	Confirmed dengue viral infection		Sensitivity	Specificity	PPV	NPV	LR+	LR–
	With shock and/or organ failure (n = 32)	No shock or organ failure (n = 128)	(95% CI)	(95% CI)	(95% CI)	(95% CI)	(95% CI)	(95% CI)
PCT (ng/mL)								
≥0.5	18	31	56.2 (37.6–73.6)	75.8 (67.4–82.9)	36.7 (23.4–51.7)	87.4 (79.7–92.9)	2.3 (1.2–3.6)	0.6 (0.4–0.9)
≥0.6	14	20	43.8 (26.4–62.3)	84.4 (76.9–90.2)	41.2 (24.6–59.3)	85.7 (78.4–91.3)	2.8 (1.6–4.9)	0.7 (0.5–0.9)
≥0.7	13	18	40.6 (23.7–59.4)	85.9 (78.7–91.4)	41.9 (24.6–60.9)	85.3 (78.0–90.9)	2.9 (1.6–5.3)	0.7 (0.5–0.9)
≥0.8	10	15	31.2 (16.1–50.0)	88.3 (81.4–93.3)	40.0 (21.1–61.3)	83.7 (76.4–89.5)	2.7 (1.3–5.4)	0.8 (0.6–1.0)
PVL (mmol/L)								
≥1.5	26	57	81.2 (63.6–92.8)	55.5 (46.4–64.2)	31.3 (21.6–42.4)	92.2 (83.8–97.1)	1.8 (1.4–2.4)	0.3 (0.2–0.7)
≥2.0	18	21	56.2 (37.7–73.6)	83.6 (76.0–89.6)	46.2 (30.1–62.8)	88.4 (81.4–93.5)	3.4 (2.1–5.6)	0.5 (0.4–0.8)
≥2.5	18	5	56.2 (37.7–73.6)	96.1 (91.1–98.7)	78.3 (56.3–92.5)	89.8 (83.4–94.3)	14.4 (5.8–35.8)	0.5 (0.3–0.7)
≥3.0	12	2	37.5 (21.1–56.3)	98.4 (94.5–99.8)	85.7 (57.2–98.2)	86.3 (79.6–91.4)	24.0 (5.6–101.9)	0.6 (0.5–0.8)
PCT (ng/mL) and/or PVL (mmol/L)								
PCT ≥0.7 and/or PVL ≥2.5	26	20	81.2 (63.6–92.8)	84.4 (76.9–90.2)	56.5 (41.1–71.1)	94.7 (88.9–98.0)	5.2 (3.4–8.0)	0.2 (0.1–0.5)

CI, confidence interval; LR+, positive likelihood ratio; LR-, negative likelihood ratio; NPV, negative predictive value; PCT, procalcitonin; PPV, positive predictive value; PVL, peripheral venous lactate.

In order to accurately predict dengue shock and/or organ failure in a greater number of patients, the optimal levels of PCT ≥0.7 ng/mL and PVL ≥2.5 mmol/L were combined as a bioscore. The AUROC for a combined bioscore in the prediction of dengue shock and/or organ failure was 0.83 (95% CI: 0.74–0.92) (Fig 3C). The combined bioscore provided good prognostic value for the prediction of dengue shock and/or organ failure among hospitalized adults with dengue, giving an optimal sensitivity of 81.2% (95% CI: 63.6–92.8%); specificity of 84.4% (95% CI: 76.9–90.2%); PPV of 56.5% (95% CI: 41.1–71.1%); NPV of 94.7% (95% CI: 88.9–98.0%); LR+ of 5.2 (95% CI: 3.4–8.0); and LR–of 0.2 (95% CI: 0.1–0.5) (Table 5 and S3 Table).

### Diagnostic values of the WHO 2009 warning signs for identifying dengue shock and/or organ failure

In order to evaluate the use of WHO 2009 WSs for identifying dengue shock and/or organ failure at admission, the diagnostic values of individual WSs and number of WSs were evaluated (S4 Table and Table 6). The sensitivities for all individual WSs were low, except for the following: lethargy, 87.5% (95% CI: 71.0–96.5%); mucosal bleeding, 78.1% (95% CI: 60.0–90.7%); and hematocrit >2% with platelets ≤100 ×10^3^/μL, 79.4% (95% CI: 40.6–76.3%). Similarly, the specificities for all WSs were low, except that for clinical fluid accumulation (89.8%; 95% CI: 83.3–94.5%). The PPVs were low, but the NPVs for the WSs were high. The LR+ values for the WSs were low, except that for clinical fluid accumulation (5.2; 95% CI: 2.8–9.6). The LR–values for the WSs ranged from 0.4 to 0.8 (S4 Table). When the number of WSs was used to identify dengue shock and/or organ failure, this resulted in an AUROC of 0.77 (95% CI: 0.68–0.87) (Fig 3D). WSs ≥4 had an optimal sensitivity of 75.0% (95% CI: 56.6–88.5%) and a specificity of 71.1% (95% CI: 62.4–78.8%). A low PPV of 39.3% (95% CI: 27.1–52.7%) and an LR+ of 2.6 (95% CI: 1.8–3.6), but a high NPV of 91.9% (95% CI: 84.7–96.4%) and an LR–of 0.4 (95% CI: 0.2–0.6), were obtained for WSs ≥4, which indicated a small decrease in the likelihood of developing dengue shock and/or organ failure when the number of WSs was <4 (Table 6).

**Table 6:** Diagnostic values of the number of WHO 2009 warning signs for identifying dengue shock and/or organ failure at admission.

Number of warning signs	Confirmed dengue viral infection		Sensitivity	Specificity	PPV	NPV	LR+	LR–
	With shock and/or organ failure (n = 32)	No shock or organ failure (n = 128)	(95% CI)	(95% CI)	(95% CI)	(95% CI)	(95% CI)	(95% CI)
≥ 2	31	102	96.9 (83.8–99.9)	20.3 (13.7–28.3)	23.3 (16.4–31.4)	96.3 (81.0–99.9)	1.2 (1.1–1.4)	0.2 (0.0–1.1)
≥ 3	26	72	81.2 (63.6–92.8)	43.8 (35.0–52.8)	26.5 (18.1–36.4)	90.3 (80.1–96.4)	1.4 (1.2–1.8)	0.4 (0.2–0.9)
≥ 4	24	37	75.0 (56.6–88.5)	71.1 (62.4–78.8)	39.3 (27.1–52.7)	91.9 (84.7–96.4)	2.6 (1.8–3.6)	0.4 (0.2–0.6)
≥ 5	21	24	65.6 (46.8–81.4)	81.2 (73.4–87.6)	46.7 (31.7–62.1)	90.4 (83.5–95.1)	3.5 (2.3–5.4)	0.4 (0.3–0.7)
≥ 6	12	7	37.5 (21.1–56.3)	94.5 (89.1–97.8)	63.2 (38.4–83.7)	85.8 (79.0–91.1)	6.9 (2.9–16.0)	0.7 (0.5–0.9)

CI, confidence interval; LR+, positive likelihood ratio; LR-, negative likelihood ratio; NPV, negative predictive value; PPV, positive predictive value; WHO, World Health Organization.

### Daily changes in serum procalcitonin and peripheral venous lactate during hospitalization among patients with or without dengue shock and/or organ failure

**Fig 4:**
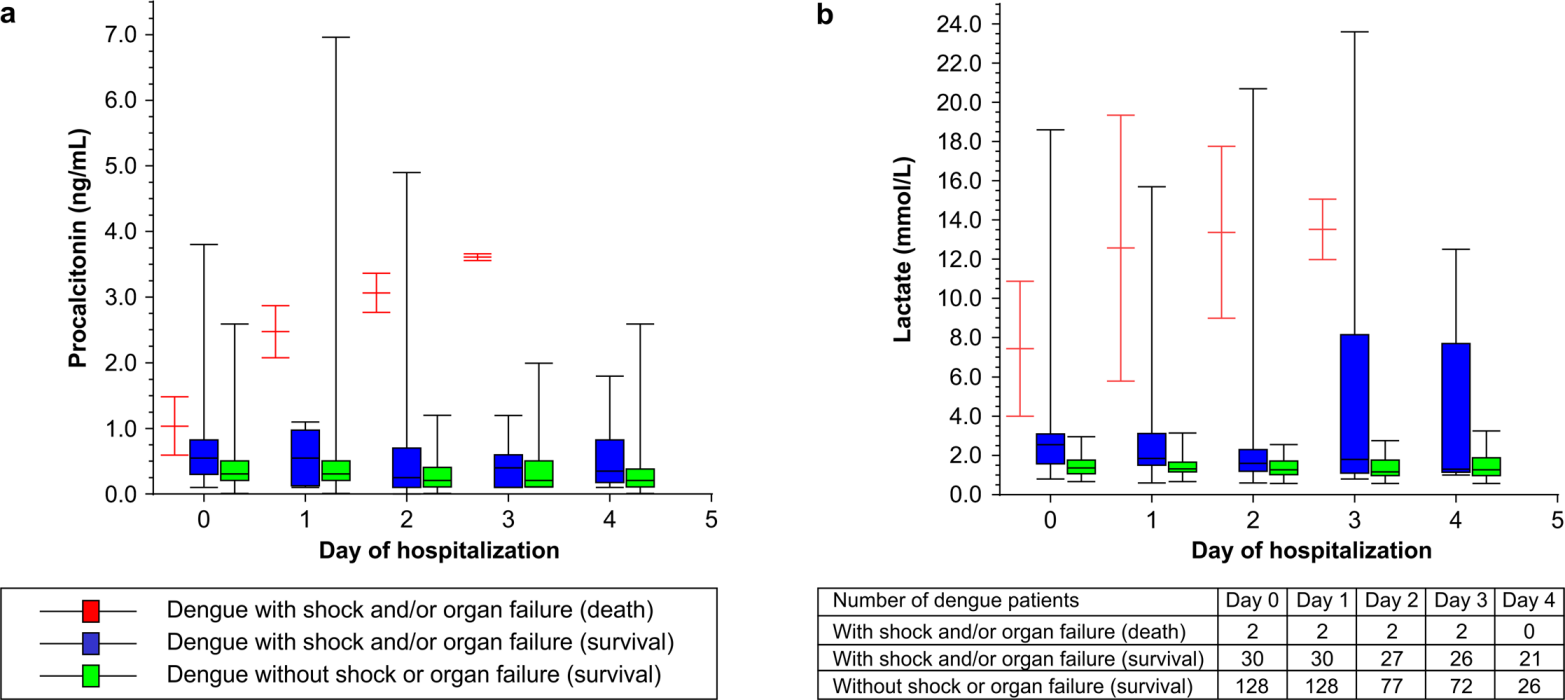
Daily changes in serum procalcitonin and peripheral venous lactate during hospitalization. (A) Changes in average serum procalcitonin (ng/mL) levels among patients with and without dengue shock and/or organ failure by survival status. (B) Changes in peripheral venous lactate (mmol/L) levels among patients with and without dengue shock and/or organ failure by survival status. Data are presented as box and whisker plots with median (horizontal line), interquartile range (box), and maximum value within 1.5 of interquartile range (whiskers).

In order to evaluate the average changes in PCT and PVL during hospitalization, PCT and PVL levels were measured at admission and every 24 h during hospitalization until the patient exhibited a body temperature <37.8°C for 48 h. Of the 32 patients who developed dengue shock and/or organ failure, only two (6.2%) expired during hospitalization, while 30 (93.8%) patients survived with complete recovery of organ function. All 128 patients without dengue shock or organ failure survived. Among the patients who survived, those with dengue shock and/or organ failure had higher PCT and PVL levels at admission and during hospitalization than those without (Fig 4). However, the patients who expired during hospitalization showed a trend toward increased PCT (>2 ng/mL) and PVL (>10 mmol/L) levels 24 h after admission (Fig 4).

## Discussion

A previous study showed that several factors were associated with dengue shock, including neurological signs, gastrointestinal bleeding, ascites, pleural effusion, hypoalbuminemia, hypoproteinemia, hepatomegaly, high levels of liver enzymes, and abnormal coagulators. It was postulated that this may have been due to the multiple organ involvement characteristic of dengue shock [13]. Recently, understanding of the pathogenesis of severe dengue has improved. It is now understood that a complex interplay between host factors and DENV is involved, resulting in vascular endothelial cell damage, which is the key factor leading to plasma leakage among patients with dengue [9–11,32]. Failure to recognize plasma leakage or inappropriate fluid administration in dengue patients with plasma leakage can lead to shock and organ failure [11,32].

Therefore, this prospective observational study was conducted among hospitalized adults with dengue in order to determine the independent factors associated with dengue shock and/or organ failure. Our results showed that PCT ≥0.7 ng/mL and PVL ≥2.5 mmol/L were independently associated with dengue shock and/or organ failure. The combination of PCT ≥0.7 ng/mL and PVL ≥2.5 mmol/L as a bioscore of 1 or 2 effectively predicted dengue shock and/or organ failure with ORs of 22.23 and 30.00, respectively. In addition, the combined bioscore provided good prognostic value in the prediction of dengue shock and/or organ failure, with an AUROC of 0.83 and an optimum sensitivity of 81.2%, specificity 84.4%, PPV 56.5%, NPV 94.7%, LR+ 5.2, and LR– 0.2. Furthermore, the combined bioscore provided a better diagnostic value for predicting dengue shock and/or organ failure compared to the WHO 2009 WSs in our study. Previous studies also showed the WSs have low sensitivities but high NPVs for identifying severe dengue in adults [33–35].

To date, PCT has been assessed as a biomarker for local and systemic inflammatory responses, disease severity, and necrosis related to organ failure, particularly in patients with bacterial infection [14,16,36]. However, a number of previous studies have shown that patients with viral diseases had PCT levels <0.5 ng/mL [37,38]. In our study, PCT ≥0.7 ng/mL was independently associated with dengue shock and/or organ failure. Previous studies have shown that patients with severe manifestations of dengue, including dengue hemorrhagic fever and dengue shock syndrome, had significantly higher viral titers than patients with dengue fever alone [39,40]. An increase in infected cells results in elevated acute-phase response proteins, cytokines, chemokines, generation of immune complexes, and consumption of complement, leading to damage of the vascular endothelium and increased vascular permeability [11,32]. PCT is an immunologically active protein induced through different steps of activation. Unlike various cytokines, PCT is activated in a time-dependent process, followed by adhesion and intercellular contact between injured cells and monocytes facilitated by adhesion molecule expression [14,15]. It is probable that increased PCT levels during DENV infection might be due to widespread inflammation in multiple organs. After cellular injury, PCT can be detected rapidly in the bloodstream within 2–6 h and reaches significant concentrations after 6 h, with peak values occurring at 12–48 h. The half-life of PCT is approximately 20–24 h with a daily clearance rate of 30% [14,41]. The clearance of PCT is not influenced by age, sex, or renal function [41].

A previous report showed that a PCT level of 0.79 ng/mL was observed among patients with localized bacterial infections [42]. In dengue, concurrent bacterial infections were observed after a median duration of 6.5 days in 4–25% of adults, and were particularly prevalent among patients with severe plasma leakage [43–45]. Sources of concurrent bacterial infection among patients with dengue included urinary tract infection (39.1%), pneumonia (38.2%), and primary bacteremia (22.7%) [43]. At admission, no patients with dengue in our study revealed bacterial growth in either of the two hemoculture samples, and there were no clinical symptoms indicative of any mixed infections. During hospitalization, four patients developed hospital-acquired infections, including two (1.6%) without dengue shock or organ failure who developed urinary tract infections, and two (6.2%) with dengue shock and/or organ failure who developed catheter-related infections during management at the intensive care unit. However, a significant proportion of patients with dengue shock and/or organ failure received antibiotics during hospitalization. In our clinical practice, antibiotics were prescribed to dengue patients suspected of having a concurrent bacterial infection, such as those with signs of peritonitis, fever with elevated bands of neutrophils, or a fever duration of more than 6 days. It is probable that bacterial translocation from the gastrointestinal or respiratory tract among patients with severe plasma leakage resulted in inflammatory responses, and increased PCT levels in the early stages of concurrent bacterial infection. A previous systematic review and meta-analysis showed that PCT ≥0.5 ng/mL could be used as a prognostic biomarker for bacterial infection, and elevated PCT levels with non-clearance were strongly associated with the in-hospital mortality of septic patients [36]. Regarding viral diseases, a previous report showed that two patients who died from 2009 H1N1 influenza infection had significantly higher mean PCT levels on admission compared to those who survived (14.5 vs. 1.7 ng/mL) [20]. Similarly, our study showed that patients who died tended to have increased PCT levels of >2 ng/mL 24 h after admission. However, the PCT levels among patients with dengue shock and/or organ failure ranged from mild to moderate elevation. The PCT assay using the Roche system in our study was previously evaluated for analytical performance in line with Clinical and Laboratory Standards. The PCT assay demonstrated acceptable precision, no evidence of nonlinearity, sample carryover or drift, and achieved high recovery from serum samples taken from patients with lower respiratory tract infections [46]. As the assay is marketed to reliably detect PCT concentrations as low as 0.02 ng/mL, the clinical laboratories providing testing are required to participate in regular international quality assurance programs to validate the test, particularly for standard hospital care.

In our study, PVL ≥2.5 mmol/L was independently associated with dengue shock and/or organ failure, regardless of hypotension. In sepsis, lactate is a biomarker of anaerobic tissue metabolism resulting from hypoxemia and hypoperfusion, and aerobic mitochondrial dysfunction termed cytopathic hypoxia [14,47,48]. Like sepsis, cytopathic hypoxia has been demonstrated during DENV infection [49,50]. In combination, PCT and PVL provided the optimal prognostic value, with a sensitivity of 81.2% and a specificity of 84.4%. These findings may have resulted from the early stage of an extensive inflammatory response during the systemic phase of dengue, or possibly the early stage of a concurrent bacterial infection. In contrast, PVL is indicative of the condition of tissue hypoperfusion before the occurrence of shock in dengue. Our study also showed that expired patients exhibited a trend toward increased PVL levels of >10 mmol/L 24 h after admission. A previous study showed that lactate clearance ≥50% during the first 6 h was an independent predictor of survival in patients with septic shock [51].

### Conclusions

The strengths of our study were the prospective observational design and assessment of serial samples for PCT and PVL analysis. In addition, treating physicians and investigators were blinded to results in order to reduce missing data and minimize bias. All participants in this study were enrolled during the febrile phase of dengue; thus, the predictive parameters determined in this study could help physicians with early prediction of dengue shock and organ failure during the critical phase of dengue. However, our study had some limitations, as follows: (1) this study was conducted in a single center in Thailand, which was the referral center for tropical infectious diseases; (2) we could not perform cultures from sites requiring invasive investigation, such as peritoneal fluid or pleural fluid, as patients with dengue are at risk of bleeding; (3) empiric antibiotics were prescribed after hemocultures were taken, and (4) although all adult patients with clinical dengue were enrolled as described in the inclusion criteria, a number of older patients with dengue do not exhibit the full range of symptoms and may therefore have been inadvertently excluded. Therefore, our study focused on the assessment of younger adults with dengue. The utility of PCT and PVL in older patients with dengue remains unknown.

Nonetheless, this study was the first to demonstrate that PCT levels ≥0.7 ng/mL and PVL levels ≥2.5 mmol/L were independently associated with dengue shock and/or organ failure, and that their combination provided good prognostic value for predicting dengue shock and/or organ failure. Dengue shock patients with non-clearance of PCT or PVL expired during hospitalization. These finding may help clinicians to predict dengue shock and/or organ failure earlier among hospitalized adults with dengue, leading to improved patient management and reduced in-hospital mortality and morbidity among patients with dengue.

## Supporting Information

S1 Checklist STROBE checklist. (DOCX)

S2 Checklist STARD checklist. (DOCX)

S1 Table Baseline characteristics and clinical parameters at admission among 160 hospitalized adults with dengue. Data are presented as median (interquartile range) unless otherwise noted.
(DOCX)

S2 Table Laboratory parameters, management, and outcomes among 160 hospitalized adults with dengue. Data are presented as median (interquartile range) unless otherwise noted. ^a^Positive serological response to infection (n = 130). ALT, alanine aminotransferase; AST, aspartate aminotransferase; LYM, lymphocytes; PMN, polymorphonuclear leukocytes; RT-PCR, reverse-transcriptase polymerase chain reaction; WBC, white blood cell counts.
(DOCX)

S3 Table Prediction of dengue shock and/or organ failure by serum procalcitonin and peripheral venous lactate levels at admission. CI, confidence interval; LR+, positive likelihood ratio; LR-, negative likelihood ratio; NPV, negative predictive value; PCT, procalcitonin; PPV, positive predictive value; PVL, peripheral venous lactate.
(DOCX)

S4 Table Diagnostic values of the WHO 2009 warning signs for identifying dengue shock and/or organ failure at admission. CI, confidence interval; LR+, positive likelihood ratio; LR-, negative likelihood ratio; NPV, negative predictive value; PPV, positive predictive value; WHO, World Health Organization.
(DOCX)
