# Supplementary material for: Serum Procalcitonin and Peripheral Venous Lactate for Predicting Dengue Shock and/or Organ Failure: A Prospective Observational Study
Source: PLoS Negl Trop Dis. 2016 Aug 26;10(8):e0004961. doi: 10.1371/journal.pntd.0004961 (PMC5001649; doi:10.1371/journal.pntd.0004961)
Supplement: S1 Checklist — (DOCX) [file pntd.0004961.s001.docx]

**Checklist S1. STROBE Statement—checklist of items that should be included in reports of observational studies**

|  | Item No. | Recommendation | Page  No. | Relevant text from manuscript |
| --- | --- | --- | --- | --- |
| **Title and abstract** | 1 | (*a*) Indicate the study’s design with a commonly used term in the title or the abstract | p1 | **Lines 1–2:** Serum Procalcitonin and Peripheral Venous Lactate for Predicting Dengue Shock and/or Organ Failure: A Prospective Observational Study |
|  |  | (*b*) Provide in the abstract an informative and balanced summary of what was done and what was found | p2 | **Lines 30–34:** A prospective observational study was conducted among adults hospitalized for confirmed viral dengue infection at the Hospital for Tropical Diseases in Bangkok, Thailand, between October 2013 and July 2015. Data, including baseline characteristics, clinical parameters, laboratory findings, serum PCT and PVL levels, management, and outcomes, were recorded on pre-defined case report forms.  **Lines 34–43:** Of 160 patients with dengue, 128 (80.0%) patients had dengue without shock or organ failure, whereas 32 (20.0%) patients developed dengue with shock and/or organ failure. Using a stepwise multivariate logistic regression analysis, PCT ≥0.7 ng/mL (odds ratio [OR]: 4.80; 95% confidence interval [CI]: 1.60–14.45; *p* = 0.005) and PVL ≥2.5 mmol/L (OR: 27.99, 95% CI: 8.47–92.53; *p* <0.001) were independently associated with dengue shock and/or organ failure. The combination of PCT ≥0.7 ng/mL and PVL ≥2.5 mmol/L provided good prognostic value for predicting dengue shock and/or organ failure, with a sensitivity of 81.2% (95% CI: 63.6–92.8%) and a specificity of 84.4% (95% CI: 76.9–90.2%). Dengue shock patients with non-clearance of PCT and PVL expired during hospitalization. |
| Introduction |  |  |  |  |
| Background/rationale | 2 | Explain the scientific background and rationale for the investigation being reported | p6 | **Lines 115–130:** Previous reports have also shown that PCT levels in patients with sepsis are associated with the severity of organ dysfunction [18], and that PCT could be used as a prognostic marker for discrimination between patients with and without septic shock, in addition to survival [19]. A previous study showed that PCT levels on admission were significantly higher among patients who died following infection with the 2009 H1N1 strain of influenza, compared with those who survived (14.5 vs. 1.7 ng/mL) [20]. In addition, arterial or venous lactate may be used as a biomarker for tissue hypoperfusion, regardless of organ failure or shock, particularly among patients with sepsis [21]. Our previous prospective study showed that peripheral venous lactate (PVL) concentration was independently associated with severe dengue [22].  In clinical practice, it can be difficult to identify the early stages of dengue shock and/or organ failure using clinical data. PCT and/or PVL may provide a superior prognostic method for predicting dengue severity at the time of hospital admission, particularly in the identification of patients at high risk of developing dengue shock and/or organ failure. At present, there have been no studies assessing the capacity of PCT and/or PVL to predict dengue shock and/or organ failure. |
| Objectives | 3 | State specific objectives, including any prespecified hypotheses | p6 | **Lines 130–135:** Thus, we hypothesized that PCT and/or PVL may discriminate between patients who develop dengue shock and/or organ failure and those who do not. Therefore, we undertook a prospective observational study among hospitalized adults with dengue and determined the factors associated with dengue shock and/or organ failure. The prognostic values of PCT and PVL as biomarkers for predicting dengue shock and/or organ failure were evaluated. |
| Methods |  |  |  |  |
| Study design | 4 | Present key elements of study design early in the paper | p7 | **Lines 153:** This prospective observational study was performed….. |
| Setting | 5 | Describe the setting, locations, and relevant dates, including periods of recruitment, exposure, follow-up, and data collection | p7,8 | **Lines 153–155:** ….patients who were admitted to the Hospital for Tropical Diseases (Faculty of Tropical Medicine, Mahidol University in Bangkok, Thailand) between October 2013 and July 2015.  **Lines 174–176:** All patient data, including baseline characteristics, clinical parameters, laboratory findings, management, and outcomes, were recorded on a pre-defined case report form. |
| Participants | 6 | (*a*) *Cohort study*—Give the eligibility criteria, and the sources and methods of selection of participants. Describe methods of follow-up  *Case-control study*—Give the eligibility criteria, and the sources and methods of case ascertainment and control selection. Give the rationale for the choice of cases and controls  *Cross-sectional study*—Give the eligibility criteria, and the sources and methods of selection of participants | p7,8 | **Lines 144–149:** Patients aged ≥15 years with clinical dengue, defined as acute fever and ≥2 of the following symptoms were included: 1) headache, 2) ocular pain, 3) myalgia, 4) arthralgia, 5) rash, 6) a positive tourniquet test (≥20 petechiae per square inch), or 7) leukopenia (white blood cell counts [WBC] <5.0 × 10^3^ cells/μL). Patients had been admitted to hospital for treatment, and the broad criteria allowed physicians to invite all potential patients to participate in the study at the outpatient and emergency department.  **Lines 155–163:** The inclusion criteria were (i) age ≥15 years, (ii) clinical dengue, and (iii) confirmed dengue viral infection by reverse-transcriptase polymerase chain reaction (RT-PCR) from a serum sample obtained at admission, and/or positive micro-neutralization test results from serum samples obtained at admission and 2 weeks after admission, and/or dengue-specific immunoglobulin M (IgM) and immunoglobulin G (IgG) detected using enzyme-linked immunosorbent assays (ELISAs) of paired serum samples taken at admission and 2 weeks after admission. Patients with an underlying medical illness, mixed infection, current pregnancy, current use of any non-topical antibiotic, or current fluid therapy were excluded from this study.  **Lines 176–179:** At a 2-week follow-up appointment, blood samples were collected for complete blood counts and serum creatinine assessment. Subsequent follow-up was required within the following 2 months until the laboratory results reached reference ranges in order to serve as a baseline. |
|  |  | (*b*) *Cohort study*—For matched studies, give matching criteria and number of exposed and unexposed  *Case-control study*—For matched studies, give matching criteria and the number of controls per case |  | n/a |
| Variables | 7 | Clearly define all outcomes, exposures, predictors, potential confounders, and effect modifiers. Give diagnostic criteria, if applicable | p8,9 | **Lines 180–192:** The WHO 2009 dengue definition was used to classify dengue shock and organ failure in this study [1]. Dengue shock was defined as plasma leakage with shock. Plasma leakage was defined as ≥20% increase in hematocrit above baseline or clinical fluid accumulation manifested by pleural effusion, ascites, or serum albumin <3.5 g/dL. Shock was defined as (1) a rapidly weak pulse with pulse pressure <20 mmHg, or (2) a systolic blood pressure of <90 mmHg with tissue hypoperfusion evidenced by one of the following criteria: (i) decreased urine output (<0.5 mL/kg/h), (ii) impaired consciousness, (iii) AST >1,000 IU/L, (iv) ALT >1,000 IU/L, (v) cold skin, or (vi) clammy skin. Organ failure was defined as the presence of one of the following criteria: (i) respiratory distress (a respiratory rate of ≥24 breaths/min with <95% oxygen saturation in room air and/or the need for oxygen therapy), (ii) serum creatinine increased ≥3-fold from baseline, (iii) AST >1,000 IU/L, (iv) ALT >1,000 IU/L, (v) myocarditis, (vi) encephalitis, or (vii) spontaneous gastrointestinal bleeding requiring blood transfusion. |
| Data sources/ measurement | 8 | For each variable of interest, give sources of data and details of methods of assessment (measurement). Describe comparability of assessment methods if there is more than one group | p8 | **Lines 164–167:** Laboratory tests were conducted at admission, including a complete blood count and blood chemistry assessment, and samples for the measurement of PCT and PVL were collected. Blood samples for PCT and PVL analysis were collected every 24 h until the patient exhibited a body temperature of <37.8ºC for 48 h.  **Lines 173–174:** Dengue severity and outcomes were summarized on discharge. |
| Bias | 9 | Describe any efforts to address potential sources of bias | p7,8 | **Lines 153–155:** This prospective observational study was performed among patients who were admitted to the Hospital for Tropical Diseases (Faculty of Tropical Medicine, Mahidol University in Bangkok, Thailand) between October 2013 and July 2015.  **Lines 170–173:** In order to exclude other infections, two blood samples for microbiological cultures were obtained, urinalysis was performed, and plain radiography of the chest was routinely performed at admission. Diagnostic tests for other infectious diseases were also performed when indicated by clinical findings at admission or during hospitalization. |
| Study size | 10 | Explain how the study size was arrived at | p11 | **Lines 249–253:** A previous prospective study at the Hospital for Tropical Diseases (Bangkok, Thailand) indicated that the incidence of dengue shock and/or organ failure was 21.0% among hospitalized adults with dengue [30]. Based on this information, we calculated that a sample size of at least 122 patients was needed for this study, using a specificity of 90% with a confidence interval (CI) of ±6%. |
| Quantitative variables | 11 | Explain how quantitative variables were handled in the analyses. If applicable, describe which groupings were chosen and why | p11,12 | **Lines 256–258:** Numerical variables were tested for normality using Kolmogorov-Smirnov tests. Variables with non-normal distribution were summarized as medians and interquartile ranges (IQRs), and were compared using Mann-Whitney *U* tests for two-group comparisons. |
| Statistical methods | 12 | (*a*) Describe all statistical methods, including those used to control for confounding | p12 | **Lines 258–265:** Categorical variables were expressed as frequencies and percentages, and were analyzed using chi-squared or Fisher’s exact tests, as appropriate. A univariate logistic regression analysis was performed with each potential factor included as an independent variable, and the presence or absence of dengue shock and/or organ failure as the dependent variable. Any variable with a *p*-value <0.2 was considered potentially significant and was further analyzed in a stepwise multivariate logistic regression analysis using a backward selection method for determining significant independent factors. |
|  |  | (*b*) Describe any methods used to examine subgroups and interactions | p12 | **Lines 269–276:** The optimal PCT and PVL cut-off values were then combined in a single “bioscore”, as described by Gibot *et al*, 2012 [31]. The bioscore attributed one point per biomarker with a value above or equal to the optimal cut-off value. The bioscore was defined as 0 (both biomarkers below their respective cut-off value), 1 (any one of the two biomarkers above/equal to the cut-off value), or 2 (both biomarkers above/equal to the cut-off value). The bioscore was then further tested for prognostic value in predicting dengue shock and/or organ failure by logistic regression analysis. |
|  |  | (*c*) Explain how missing data were addressed |  | n/a |
|  |  | (*d*) *Cohort study*—If applicable, explain how loss to follow-up was addressed  *Case-control study*—If applicable, explain how matching of cases and controls was addressed  *Cross-sectional study*—If applicable, describe analytical methods taking account of sampling strategy |  | n/a |
|  |  | (*e*) Describe any sensitivity analyses |  | n/a |
| **Results** |  |  |  |  |
| Participants | 13 | (a) Report numbers of individuals at each stage of study—eg numbers potentially eligible, examined for eligibility, confirmed eligible, included in the study, completing follow-up, and analysed | p13 | **Lines 281–286:** A total of 189 adults with suspected dengue were admitted to the Hospital for Tropical Diseases (Bangkok, Thailand) between October 2013 and July 2015. Of 189 hospitalized adults with suspected dengue viral infection, 29 patients were excluded due to an underlying illness (17 patients, 58.6%), mixed infection (10 patients, 34.5%), or a negative RT-PCR/micro-neutralization/ELISA for dengue (2 patients, 6.9%). Thus, 160 hospitalized adults with confirmed dengue viral infection were finally recruited for this study. |
|  |  | (b) Give reasons for non-participation at each stage | p13 | **Lines 282–285:** Of 189 hospitalized adults with suspected dengue viral infection, 29 patients were excluded due to an underlying illness (17 patients, 58.6%), mixed infection (10 patients, 34.5%), or a negative RT-PCR/micro-neutralization/ELISA for dengue (2 patients, 6.9%). |
|  |  | (c) Consider use of a flow diagram | p13 | **Lines 286–289:** Among the 160 patients, 32 (20.0%) patients had dengue shock (23 patients [71.9%]) and/or organ failure (26 patients [81.2%]), whereas 128 (80.0%) patients had dengue without shock or organ failure (Fig 1). |
| Descriptive data | 14 | (a) Give characteristics of study participants (eg demographic, clinical, social) and information on exposures and potential confounders | p13,14 | **Lines 300–307:** At admission, patients with dengue shock and/or organ failure were significantly more likely to have a longer duration of fever (*p* = 0.031), skin bleeding (*p* = 0.012), mucosal bleeding (*p* <0.001), vomiting (*p* = 0.024), a liver span of >15 cm (*p* = 0.001), decreased breathing sounds (*p* <0.001), and increased respiratory rate (*p* = 0.010). When numerical parameters were categorized, patients aged >40 years (*p* = 0.023), with a fever duration ≥5 days (*p* = 0.041), respiratory rate ≥24 breaths/min (*p* = 0.005), mean arterial pressure <70 mmHg (*p* = 0.030), or pulse pressure <30 mmHg (*p* = 0.005) were more likely to have dengue shock and/or organ failure (Table 1 and Table S1). |
|  |  | (b) Indicate number of participants with missing data for each variable of interest |  | n/a |
|  |  | (c) *Cohort study*—Summarise follow-up time (eg, average and total amount) | p15 | **Lines 334–341:** Assessment of patient management and outcomes during hospitalization demonstrated that a significant proportion of patients with dengue shock and/or organ failure received albumin as fluid resuscitation (*p* <0.001) and antibiotics (*p* = 0.017). Of the 32 patients with dengue shock and/or organ failure, 4 (12.5%) received mechanical ventilation, 3 (9.4%) received renal replacement therapy, and 2 (6.2%) received vasopressors. Patients with dengue shock and/or organ failure had significantly longer durations of hospitalization (*p* = 0.006). However, only two patients expired during hospitalization, both due to multi-organ failure (Table S2). |
| Outcome data | 15 | *Cohort study*—Report numbers of outcome events or summary measures over time | p13 | **Lines 286–289:** Among the 160 patients, 32 (20.0%) patients had dengue shock (23 patients [71.9%]) and/or organ failure (26 patients [81.2%]), whereas 128 (80.0%) patients had dengue without shock or organ failure (Fig 1). |
|  |  | *Case-control study—*Report numbers in each exposure category, or summary measures of exposure |  | n/a |
|  |  | *Cross-sectional study—*Report numbers of outcome events or summary measures |  | n/a |
| Main results | 16 | (*a*) Give unadjusted estimates and, if applicable, confounder-adjusted estimates and their precision (eg, 95% confidence interval). Make clear which confounders were adjusted for and why they were included | p16,17 | **Lines 352–364:** All clinical factors potentially associated with the occurrence of dengue shock and/or organ failure were included in the univariate logistic regression analysis. The following variables were identified as clinical parameters associated with dengue shock and/or organ failure: (1) age >40 years, (2) fever duration ≥5 days, (3) absolute bands >200 cells/μL, (4) absolute atypical lymphocyte counts >300 cells/μL, (5) PCT ≥0.7 ng/mL, and (6) PVL ≥2.5 mmol/L (Table 3).  All parameters with a p-value ≤0.2 in the univariate logistic regression analysis were then further analyzed by a stepwise multivariate logistic regression analysis using a backward selection method, in order to determine the independent factors significantly associated with the occurrence of dengue shock and/or organ failure. The following clinical and laboratory parameters were found to be independently associated with the occurrence of dengue shock and/or organ failure: (1) PCT ≥0.7 ng/mL (odds ratio [OR]: 4.80; 95% CI: 1.60–14.45; *p* = 0.005) and (2) PVL ≥2.5 mmol/L (OR: 27.99, 95% CI: 8.47–92.53; *p* <0.001) (Table 4). |
|  |  | (*b*) Report category boundaries when continuous variables were categorized | p14,15 | **Lines 312–325:** Regarding laboratory parameters (Table 2 and Table S2), patients with dengue shock and/or organ failure had significantly higher hemoglobin concentrations (*p* = 0.045), increased hematocrit values above baseline (*p* <0.001), higher WBC counts (*p* = 0.044), higher absolute bands (*p* = 0.022), higher absolute atypical lymphocyte counts (*p* = 0.007), higher AST levels (*p* <0.001), higher ALT levels (*p* <0.001), higher PCT levels (*p* = 0.001), and higher PVL levels (*p* <0.001) (Fig 2). However, patients with dengue shock and/or organ failure had significantly lower platelet counts (*p* <0.001) and albumin levels (*p* <0.001). When laboratory parameters were categorized based on the reference ranges (Table 2), patients with WBC counts >5.0 × 10^3^ cells/μL (*p* = 0.004), absolute bands >200 cells/μL (*p* = 0.049), absolute atypical lymphocyte counts >300 cells/μL (*p* = 0.006), AST >120 IU/L (*p* = 0.002), ALT >120 IU/L (*p* = 0.002), PCT ≥0.7 ng/mL (*p* = 0.002), and PVL ≥2.5 mmol/L (*p* <0.001) were more likely to have dengue shock and/or organ failure. In addition, patients with platelet counts <50.0 × 10^3^ cells/μL (*p* = 0.012) and albumin <3.5 g/dL (*p* = 0.001) were also more likely to have dengue shock and/or organ failure. |
|  |  | (*c*) If relevant, consider translating estimates of relative risk into absolute risk for a meaningful time period |  | n/a |
| Other analyses | 17 | Report other analyses done—eg analyses of subgroups and interactions, and sensitivity analyses | p17 | **Lines 364–370:** The two biomarkers PCT ≥0.7 ng/mL and PVL ≥2.5 mmol/L were assessed as a combined bioscore using a logistic regression model to evaluate the prognostic capacity in predicting the occurrence of dengue shock and/or organ failure. Higher bioscores were associated with increased occurrence of dengue shock and/or organ failure, with ORs of 22.23 (95% CI 7.85–63.00) and 30.00 (95% CI 5.76–156.31) for a bioscore 1 and 2, respectively (*p* <0.001) (Table 4). |
| **Discussion** |  |  |  |  |
| Key results | 18 | Summarise key results with reference to study objectives | p22 | **Lines 440–448:** Therefore, this prospective observational study was conducted among hospitalized adults with dengue in order to determine the independent factors associated with dengue shock and/or organ failure. Our results showed that PCT ≥0.7 ng/mL and PVL ≥2.5 mmol/L were independently associated with dengue shock and/or organ failure. The combination of PCT ≥0.7 ng/mL and PVL ≥2.5 mmol/L as a bioscore of 1 or 2 effectively predicted dengue shock and/or organ failure with ORs of 22.23 and 30.00, respectively. In addition, the combination of PCT ≥0.7 ng/mL and PVL ≥2.5 mmol/L provided good prognostic value in the prediction of dengue shock and/or organ failure, with an optimum sensitivity of 81.2%, specificity 84.4%, PPV 56.5%, NPV 94.7%, LR+ 5.2, and LR– 0.2. |
| Limitations | 19 | Discuss limitations of the study, taking into account sources of potential bias or imprecision. Discuss both direction and magnitude of any potential bias | p25,26 | **Lines 522–531:** However, our study had some limitations, as follows: (1) this study was conducted in a single center in Thailand, which was the referral center for tropical infectious diseases; (2) we could not perform cultures from sites requiring invasive investigation, such as peritoneal fluid or pleural fluid, as patients with dengue are at risk of bleeding; (3) empiric antibiotics were prescribed after hemocultures were taken, and (4) although all adult patients with clinical dengue were enrolled as described in the inclusion criteria, a number of older patients with dengue do not exhibit the full range of symptoms and may therefore have been inadvertently excluded. Therefore, our study focused on the assessment of younger adults with dengue. The utility of PCT and PVL in older patients with dengue remains unknown. |
| Interpretation | 20 | Give a cautious overall interpretation of results considering objectives, limitations, multiplicity of analyses, results from similar studies, and other relevant evidence | p26 | **Lines 532–538:** Nonetheless, this study was the first to demonstrate that PCT levels ≥0.7 ng/mL and PVL levels ≥2.5 mmol/L were independently associated with dengue shock and/or organ failure, and that their combination provided good prognostic value for predicting dengue shock and/or organ failure. Dengue shock patients with non-clearance of PCT or PVL expired during hospitalization. These finding may help clinicians to predict dengue shock and/or organ failure earlier among hospitalized adults with dengue, leading to improved patient management and reduced in-hospital mortality and morbidity among patients with dengue. |
| Generalisability | 21 | Discuss the generalisability (external validity) of the study results | p25,26 | **Lines 522–524:** However, our study had some limitations, as follows: (1) this study was conducted in a single center in Thailand, which was the referral center for tropical infectious diseases;  **Lines 527–531:** (4) although all adult patients with clinical dengue were enrolled as described in the inclusion criteria, a number of older patients with dengue do not exhibit the full range of symptoms and may therefore have been inadvertently excluded. Therefore, our study focused on the assessment of younger adults with dengue. The utility of PCT and PVL in older patients with dengue remains unknown. |
| **Other information** |  |  |  |  |
| Funding | 22 | Give the source of funding and the role of the funders for the present study and, if applicable, for the original study on which the present article is based | In submission information | This study was supported by the Dean’s Fund Research 2013, Faculty of Tropical Medicine, Mahidol University; Research Grant 2013, the Royal College of Physicians of Thailand; and Roche Diagnostics (Thailand) Ltd. for providing Elecsys BRAHMS PCT for this study. The funders had no role in study design, data collection and/or analysis, the decision to publish, or preparation of the manuscript. |
