# Supplementary material for: Serum Procalcitonin and Peripheral Venous Lactate for Predicting Dengue Shock and/or Organ Failure: A Prospective Observational Study
Source: PLoS Negl Trop Dis. 2016 Aug 26;10(8):e0004961. doi: 10.1371/journal.pntd.0004961 (PMC5001649; doi:10.1371/journal.pntd.0004961)
Supplement: S1 Table — Data are presented as median (interquartile range) unless otherwise noted. (DOCX) [file pntd.0004961.s003.docx]

**S1 Table.** **Baseline characteristics and clinical parameters at admission among 160 hospitalized adults with dengue.**

| Characteristic | With dengue shock and/or organ failure (n = 32) | No dengue shock or organ failure (n = 128) | *p*-value |
| --- | --- | --- | --- |
| Age (years) | 26.0 (20.0–45.8) | 23.5 (19.0–34.0) | 0.057 |
| Male sex, n (%) | 18 (56.3) | 69 (53.9) | 0.968 |
| Fever (days) | 5.0 (4.0–6.0) | 4.0 (3.0–5.0) | 0.031 |
| Myalgia, n (%) | 29 (90.6) | 116 (90.6) | 1.000 |
| Lethargy, n (%) | 28 (87.5) | 97 (75.8) | 0.232 |
| Headache, n (%) | 27 (84.4) | 108 (84.4) | 1.000 |
| Ocular pain, n (%) | 16 (50.0) | 86 (67.2) | 0.109 |
| Abdominal pain, n (%) | 16 (50.0) | 48 (37.5) | 0.276 |
| Cough, n (%) | 13 (40.6) | 50 (39.1) | 1.000 |
| Arthralgia, n (%) | 12 (37.5) | 35 (27.3) | 0.362 |
| Diarrhea, n (%) | 11 (34.4) | 39 (30.5) | 0.831 |
| Decreased breathing sounds, n (%) | 10 (31.3) | 7 (5.5) | <0.001 |
| Temperature (ºC) | 38.0 (37.2–39.2) | 38.4 (37.8–39.1) | 0.364 |
| Respiratory rate (breaths/min) | 22 (20–24) | 20 (18–22) | 0.010 |
| Heart rate (beats/min) | 81 (68–92) | 78 (66–88) | 0.309 |
| Mean arterial pressure (mmHg) | 86 (73–96) | 84 (77–91) | 0.612 |
| Pulse pressure (mmHg) | 35 (26–52) | 38 (32–44) | 0.517 |
