# Supplementary material for: Serum Procalcitonin and Peripheral Venous Lactate for Predicting Dengue Shock and/or Organ Failure: A Prospective Observational Study
Source: PLoS Negl Trop Dis. 2016 Aug 26;10(8):e0004961. doi: 10.1371/journal.pntd.0004961 (PMC5001649; doi:10.1371/journal.pntd.0004961)
Supplement: S2 Table — Data are presented as median (interquartile range) unless otherwise noted. aPositive serological response to infection (n = 130). ALT, alanine aminotransferase; AST, aspartate aminotransferase; LYM, lymphocytes; PMN, polymorphonuclear leukocytes; RT-PCR, reverse-transcriptase polymerase chain reaction; WBC, white blood cell counts. (DOCX) [file pntd.0004961.s004.docx]

**S2 Table. Laboratory parameters, management, and outcomes among 160 hospitalized adults with dengue.**

| Characteristic | With dengue shock and/or organ failure (n = 32) | No dengue shock or organ failure (n = 128) | *p*-value |
| --- | --- | --- | --- |
| Dengue RT-PCR or micro-neutralization test |  |  |  |
| Serotypes 1 or 4, n (%) | 12 (37.5) | 52 (40.6) | 0.904 |
| Serotypes 2 or 3, n (%) | 20 (62.5) | 76 (59.4) |  |
| Positive serological response to infection^a^ |  |  |  |
| Primary, n (%) | 2 (7.7) | 3 (2.9) | 0.261 |
| Secondary, n (%) | 24 (92.3) | 101 (97.1) |  |
| Hematology |  |  |  |
| Hemoglobin (g/dL) | 14.5 (12.8–16.2) | 13.8 (12.8–14.8) | 0.045 |
| Hematocrit above baseline (%) | 20.7 (7.5–29.0) | 3.8 (-0.8–9.5) | <0.001 |
| WBC (×10^3^ cells/μL) | 4.6 (2.6–6.6) | 3.4 (2.5–4.8) | 0.044 |
| Absolute bands (cells/μL) | 201 (103–368) | 126 (53–250) | 0.022 |
| Absolute PMN (cells/μL) | 1616 (1280–2870) | 1818 (1068–2590) | 0.490 |
| Absolute LYM (cells/μL) | 980 (524–1763) | 764 (546–1101) | 0.154 |
| Absolute atypical LYM (cells/μL) | 388 (93–1344) | 126 (53–405) | 0.007 |
| Platelet counts (×10^3^/μL) | 56.0 (18.5–87.2) | 94.0 (59.2–146.2) | <0.001 |
| Blood chemistries |  |  |  |
| Creatinine (mg/dL) | 0.9 (0.7–1.1) | 0.8 (0.6–1.0) | 0.052 |
| Albumin (g/dL) | 3.9 (3.4–4.3) | 4.3 (4.0–4.5) | <0.001 |
| AST (IU/L) | 229 (84–684) | 65 (35–144) | <0.001 |
| ALT (IU/L) | 108 (76–392) | 44 (18–97) | <0.001 |
| Procalcitonin (ng/mL) | 0.6 (0.3–0.9) | 0.3 (0.2–0.5) | 0.001 |
| Lactate (mmol/L) | 2.6 (1.6–3.2) | 1.4 (1.1–1.8) | <0.001 |
| Management and outcomes |  |  |  |
| Vasopressors, n (%) | 2 (6.2) | - |  |
| Renal replacement therapy, n (%) | 3 (9.4) | - |  |
| Mechanical ventilation, n (%) | 4 (12.5) | - |  |
| Albumin resuscitation, n (%) | 10 (31.3) | 2 (1.6) | <0.001 |
| Antibiotics, n (%) | 17 (53.1) | 37 (28.9) | 0.017 |
| Hospitalization (days) | 4.6 (2.9–9.5) | 3.3 (2.5–4.7) | 0.006 |
| In-hospital mortality, n (%) | 2 (6.2) | - |  |

ALT, alanine aminotransferase; AST, aspartate aminotransferase; LYM, lymphocytes; PMN, polymorphonuclear leukocytes; RT-PCR, reverse-transcriptase polymerase chain reaction; WBC, white blood cell counts.
