# Supplementary material for: Serum Procalcitonin and Peripheral Venous Lactate for Predicting Dengue Shock and/or Organ Failure: A Prospective Observational Study
Source: PLoS Negl Trop Dis. 2016 Aug 26;10(8):e0004961. doi: 10.1371/journal.pntd.0004961 (PMC5001649; doi:10.1371/journal.pntd.0004961)
Supplement: S3 Table — CI, confidence interval; LR+, positive likelihood ratio; LR-, negative likelihood ratio; NPV, negative predictive value; PCT, procalcitonin; PPV, positive predictive value; PVL, peripheral venous lactate. (DOCX) [file pntd.0004961.s005.docx]

**S3 Table. Prediction of dengue shock and/or organ failure by serum procalcitonin and peripheral venous lactate levels at admission.**

| Cut-off value | Confirmed dengue viral infection | | Sensitivity  (95% CI) | Specificity  (95% CI) | PPV  (95% CI) | NPV  (95% CI) | LR+  (95% CI) | LR–  (95% CI) |
| --- | --- | --- | --- | --- | --- | --- | --- | --- |
|  | With shock and/or organ failure (n = 32) | No shock or organ failure (n = 128) |  |  |  |  |  |  |
| PCT (ng/mL) and/or PVL (mmol/L) | | | | | | | | |
| PCT ≥0.5 and/or PVL ≥2.5 | 27 | 33 | 84.4 (67.2–94.7) | 74.2 (65.7–81.5) | 45.0 (32.1–58.4) | 95.0 (88.7–98.4) | 3.3 (2.4–4.6) | 0.2 (0.1–0.5) |
| PCT ≥0.6 and/or PVL ≥2.5 | 26 | 22 | 81.2 (63.6–92.8) | 82.8 (75.1–88.9) | 54.2 (39.2– 68.6) | 94.6 (88.7–98.0) | 4.7 (3.1–7.2) | 0.2 (0.1–0.5) |
| PCT ≥0.7 and/or PVL ≥2.5 | 26 | 20 | 81.2 (63.6–92.8) | 84.4 (76.9– 90.2) | 56.5 (41.1–71.1) | 94.7 (88.9–98.0) | 5.2 (3.4–8.0) | 0.2 (0.1–0.5) |
| PCT ≥0.8 and/or PVL ≥2.5 | 23 | 18 | 71.9 (53.2–86.2) | 85.9 (78.7–91.4) | 56.1 (39.8–71.5) | 92.4 (86.1–96.5) | 5.1 (3.2–8.3) | 0.3 (0.2–0.6) |

CI, confidence interval; LR+, positive likelihood ratio; LR-, negative likelihood ratio; NPV, negative predictive value; PCT, procalcitonin; PPV, positive predictive value; PVL, peripheral venous lactate.
