# Supplementary material for: Serum Procalcitonin and Peripheral Venous Lactate for Predicting Dengue Shock and/or Organ Failure: A Prospective Observational Study
Source: PLoS Negl Trop Dis. 2016 Aug 26;10(8):e0004961. doi: 10.1371/journal.pntd.0004961 (PMC5001649; doi:10.1371/journal.pntd.0004961)
Supplement: S4 Table — CI, confidence interval; LR+, positive likelihood ratio; LR-, negative likelihood ratio; NPV, negative predictive value; PPV, positive predictive value; WHO, World Health Organization. (DOCX) [file pntd.0004961.s006.docx]

**S4 Table. Diagnostic values of the WHO 2009 warning signs for identifying dengue shock and/or organ failure at admission.**

| Cut-off value | Confirmed dengue viral infection | | Sensitivity  (95% CI) | Specificity  (95% CI) | PPV  (95% CI) | NPV  (95% CI) | LR+  (95% CI) | LR–  (95% CI) |
| --- | --- | --- | --- | --- | --- | --- | --- | --- |
|  | With shock and/or organ failure (n = 32) | No shock or organ failure (n = 128) |  |  |  |  |  |  |
| Abdominal pain | 16 | 48 | 50.0 (31.9–68.1) | 62.5 (53.5–70.9) | 25.0 (15.0–37.4) | 83.3 (74.4–90.2) | 1.3 (0.9–2.0) | 0.8 (0.6–1.2) |
| Vomiting | 22 | 57 | 68.8 (50.0–83.9) | 55.5 (46.4–64.2) | 27.8 (18.4–39.1) | 87.6 (78.5–93.9) | 1.5 (1.1–2.1) | 0.6 (0.3–1.0) |
| Lethargy | 28 | 97 | 87.5 (71.0–96.5) | 24.2 (17.1–32.6) | 22.4 (15.4–30.7) | 88.6 (73.3–96.8) | 1.2 (1.0–1.4) | 0.5 (0.2–1.4) |
| Liver span of >15 cm | 22 | 44 | 68.8 (50.0–83.9) | 65.6 (56.7–73.8) | 33.3 (22.2–46.0) | 89.4 (81.3–94.8) | 2.0 (1.4–2.9) | 0.5 (0.3–0.8) |
| Mucosal bleeding | 25 | 52 | 78.1 (60.0–90.7) | 59.4 (50.3–68.0) | 32.5 (22.2–44.1) | 91.6 (83.4–96.5) | 1.9 (1.5–2.5) | 0.4 (0.2–0.7) |
| Clinical fluid accumulation | 17 | 13 | 53.1 (34.7–70.9) | 89.8 (83.3–94.5) | 56.7 (37.4–74.5) | 88.5 (81.7–93.4) | 5.2 (2.8–9.6) | 0.5 (0.4–0.8) |
| Hematocrit >2% and platelets ≤100 ×10^3^/μL | 19 | 49 | 79.4 (40.6–76.3) | 61.7 (52.7–70.2) | 27.9 (17.7–40.2) | 85.9 (77.0–92.3) | 1.6 (1.1–2.2) | 0.7 (0.4–1.0) |

CI, confidence interval; LR+, positive likelihood ratio; LR-, negative likelihood ratio; NPV, negative predictive value; PPV, positive predictive value; WHO, World Health Organization.
